# Supplementary material for: Propagation of viral bioaerosols indoors
Source: PLoS One. 2021 Jan 5;16(1):e0244983. doi: 10.1371/journal.pone.0244983 (PMC7785217; doi:10.1371/journal.pone.0244983)
Supplement: S1 Table — (DOCX) [file pone.0244983.s001.docx]

S1 Table. Mean aerosol particles concentration vs. time.

1. The test box of horizontal configuration (Fig 5)
   1. L-shaped configuration, atomization at one spot (A)
      1. Over sprayer

| **Time, s** | **Mean(n=10)** | **Std** | **Std/Mean,** **%** |
| --- | --- | --- | --- |
| 10 | 3.081 | 0.066 | 2.15 |
| 30 | 2.895 | 0.088 | 3.05 |
| 60 | 2.507 | 0.066 | 2.64 |
| 90 | 1.801 | 0.122 | 6.76 |
| 120 | 1.498 | 0.100 | 6.68 |
| 150 | 1.068 | 0.059 | 5.52 |
| 180 | 1.075 | 0.088 | 8.23 |
| 210 | 0.874 | 0.054 | 6.22 |
| 240 | 0.495 | 0.035 | 7.11 |
| 270 | 0.473 | 0.036 | 7.52 |
| 300 | 0.480 | 0.025 | 5.24 |

- - 1. Distance 1.5 m

| **Time, s** | **Mean(n=10)** | **Std** | **Std/Mean,** **%** |
| --- | --- | --- | --- |
| 10 | 0 | 0 |  |
| 30 | 0 | 0 |  |
| 60 | 0.007 | 0.0002 | 2.52 |
| 90 | 0.240 | 0.0077 | 3.19 |
| 120 | 0.143 | 0.0069 | 4.81 |
| 150 | 0.251 | 0.0115 | 4.58 |
| 180 | 0.183 | 0.0069 | 3.77 |
| 210 | 0.114 | 0.0022 | 1.96 |
| 240 | 0.044 | 0.0018 | 4.07 |
| 270 | 0.117 | 0.0032 | 2.77 |
| 300 | 0.132 | 0.0043 | 3.26 |

- 1. L-shaped configuration, atomization at two spots (B)
     1. Over sprayer

| **Time, s** | **Mean(n=10)** | **Std** | **Std/Mean,** **%** |
| --- | --- | --- | --- |
| 10 | 2.854 | 0.141 | 4.96 |
| 30 | 1.328 | 0.053 | 3.99 |
| 60 | 0.936 | 0.024 | 2.51 |
| 90 | 0.844 | 0.024 | 2.81 |
| 120 | 0.592 | 0.018 | 3.05 |
| 150 | 0.437 | 0.017 | 3.96 |
| 180 | 0.307 | 0.012 | 3.82 |
| 210 | 0.230 | 0.013 | 5.76 |
| 240 | 0.265 | 0.017 | 6.32 |
| 270 | 0.200 | 0.012 | 5.95 |
| 300 | 0.198 | 0.006 | 2.88 |

- - 1. Distance 1 m

| **Time, s** | **Mean(n=10)** | **Std** | **Std/Mean,** **%** |
| --- | --- | --- | --- |
| 10 | 0.050 | 0.0034 | 6.74 |
| 30 | 0.032 | 0.0026 | 8.19 |
| 60 | 0.093 | 0.0045 | 4.83 |
| 90 | 0.196 | 0.0128 | 6.56 |
| 120 | 0.163 | 0.0090 | 5.51 |
| 150 | 0.162 | 0.0047 | 2.92 |
| 180 | 0.095 | 0.0044 | 4.64 |
| 210 | 0.174 | 0.0070 | 4.02 |
| 240 | 0.158 | 0.0054 | 3.40 |
| 270 | 0.129 | 0.0047 | 3.64 |
| 300 | 0.176 | 0.0044 | 2.52 |

1. The test box of vertical configuration (Fig. 6A)
   1. Over sprayer

| **Time, s** | **Mean(n=10)** | **Std** | **Std/Mean,** **%** |
| --- | --- | --- | --- |
| 10 | 2.854 | 0.141 | 4.96 |
| 30 | 1.328 | 0.053 | 3.99 |
| 60 | 0.936 | 0.024 | 2.51 |
| 90 | 0.844 | 0.024 | 2.81 |
| 120 | 0.592 | 0.018 | 3.05 |
| 150 | 0.437 | 0.017 | 3.96 |
| 180 | 0.307 | 0.012 | 3.82 |
| 210 | 0.230 | 0.013 | 5.76 |
| 240 | 0.265 | 0.017 | 6.32 |
| 270 | 0.200 | 0.012 | 5.95 |
| 300 | 0.198 | 0.006 | 2.88 |

- 1. 1.5 m down

| **Time, s** | **Mean(n=10)** | **Std** | **Std/Mean,** **%** |
| --- | --- | --- | --- |
| 10 | 0.058 | 0.003 | 4.44 |
| 30 | 0.052 | 0.002 | 3.89 |
| 60 | 0.106 | 0.008 | 7.75 |
| 90 | 0.336 | 0.012 | 3.63 |
| 120 | 0.296 | 0.010 | 3.36 |
| 150 | 0.247 | 0.006 | 2.58 |
| 180 | 0.335 | 0.012 | 3.71 |
| 210 | 0.253 | 0.015 | 5.89 |
| 240 | 0.268 | 0.012 | 4.35 |
| 270 | 0.211 | 0.012 | 5.83 |
| 300 | 0.214 | 0.012 | 5.43 |

- 1. 1.5 m up

| **Time, s** | **Mean(n=10)** | **Std** | **Std/Mean,** **%** |
| --- | --- | --- | --- |
| 10 | 0.001 | 0.0001 | 6.52 |
| 30 | 0.002 | 0.0001 | 7.26 |
| 60 | 0.009 | 0.0003 | 3.07 |
| 90 | 0.152 | 0.0072 | 4.77 |
| 120 | 0.196 | 0.0061 | 3.09 |
| 150 | 0.190 | 0.0059 | 3.13 |
| 180 | 0.276 | 0.0113 | 4.09 |
| 210 | 0.194 | 0.0056 | 2.90 |
| 240 | 0.182 | 0.0066 | 3.64 |
| 270 | 0.184 | 0.0053 | 2.85 |
| 300 | 0.187 | 0.0092 | 4.95 |
